# Prospection of Fungal Lignocellulolytic Enzymes Produced from Jatoba (*Hymenaea courbaril*) and Tamarind (*Tamarindus indica*) Seeds: Scaling for Bioreactor and Saccharification Profile of Sugarcane Bagasse

**DOI:** 10.3390/microorganisms9030533

**Published:** 2021-03-05

**Authors:** Alex Graça Contato, Tássio Brito de Oliveira, Guilherme Mauro Aranha, Emanuelle Neiverth de Freitas, Ana Claudia Vici, Karoline Maria Vieira Nogueira, Rosymar Coutinho de Lucas, Ana Sílvia de Almeida Scarcella, Marcos Silveira Buckeridge, Roberto Nascimento Silva, Maria de Lourdes Teixeira de Moraes Polizeli

**Affiliations:** 1 Departamento de Bioquímica e Imunologia, Faculdade de Medicina de Ribeirão Preto, Universidade de São Paulo, Ribeirão Preto 14049-900, Brazil; alexgraca.contato@gmail.com (A.G.C.); emanuelleneiverthf@gmail.com (E.N.d.F.); karolmvnogueira@gmail.com (K.M.V.N.); rosymar_lucas@hotmail.com (R.C.d.L.); asascarcella@yahoo.com.br (A.S.d.A.S.); rsilvausp@gmail.com (R.N.S.); 2 Departamento de Biologia, Faculdade de Filosofia, Ciências e Letras de Ribeirão Preto, Universidade de São Paulo, Ribeirão Preto 14050-901, Brazil; oliveiratb@yahoo.com.br (T.B.d.O.); guilhermearanha@usp.br (G.M.A.); acvici@gmail.com (A.C.V.); 3 Departamento de Botânica, Instituto de Biociências, Universidade de São Paulo, São Paulo 05508-090, Brazil; msbuck@usp.br

**Keywords:** lignocellulosic biomass, bioprospecting, *Hymenaea courbaril*, *Tamarindus indica*, enzymes

## Abstract

The lignocellulosic biomass comprises three main components: cellulose, hemicellulose, and lignin. Degradation and conversion of these three components are attractive to biotechnology. This study aimed to prospect fungal lignocellulolytic enzymes with potential industrial applications, produced through a temporal analysis using *Hymenaea courbaril* and *Tamarindus indica* seeds as carbon sources. α-L-arabinofuranosidase, acetyl xylan esterase, endo-1,5-α-L-arabinanase, β-D-galactosidase, β-D-glucosidase, β-glucanase, β-D-xylosidase, cellobiohydrolase, endoglucanase, lichenase, mannanase, polygalacturonase, endo-1,4-β-xylanase, and xyloglucanase activities were determined. The enzymes were produced for eight filamentous fungi: *Aspergillus fumigatus*, *Trametes hirsuta*, *Lasiodiplodia* sp., two strains of *Trichoderma longibrachiatum*, *Neocosmospora perseae*, *Fusarium* sp. and *Thermothelomyces thermophilus*. The best producers concerning enzymatic activity were *T. thermophilus* and *T. longibrachiatum*. The optimal conditions for enzyme production were the media supplemented with tamarind seeds, under agitation, for 72 h. This analysis was essential to demonstrate that cultivation conditions, static and under agitation, exert strong influences on the production of several enzymes produced by different fungi. The kind of sugarcane, pretreatment used, microorganisms, and carbon sources proved limiting sugar profile factors.

## 1. Introduction

The lignocellulosic biomass, since ancient times, has been an essential source of energy for humanity. It is estimated that it contributes to 10–14% of the world’s energy supply. The degradation and conversion of the biomass components are attractive due to their employability in developing sustainable and environmentally clean bioenergy. It can also be applied in the biomaterials and biorefinery industries [1,2,3].

Plant cell walls, which constitute lignocellulosic biomass, consist of carbohydrate polymers like cellulose, hemicellulose, xyloglucans, arabinoxylans, and mannans, β-glucans, homogalacturonans, arabinogalactans structural proteins, and phenolic compounds. The presence of these compounds depends on the age, part, and type of plants [4,5,6]. Hemicellulose, pectin, and some structural proteins make up the cellular matrix, forming a distributive network between cellulose microfibrils [7]. Their function is to confer mechanical and chemical resistance against pathogens and deteriorating agents [8].

Due to the constitution, seeds of tamarind (*Tamarindus indica* Linn.) and jatoba (*Hymenaea courbaril*) may be used for the growth of microorganisms or substrates for microbial enzyme production with application in hydrolysis of biomass. The use of tamarind as a substrate for lignocellulolytic enzyme production with industrial potential has already been reported [9,10]. Its seeds, waste from the fruit pulp industry, are rich in phytochemicals and proteins [11]. Jatoba was originated in Africa about 65 million years ago [12]. It has large seeds with non-photosynthetic globular cotyledons, rich in a cell wall polysaccharide (xyloglucan), which corresponds to about 40% of the seed dry mass [13,14]. The use of seeds of these plants like a substrate is already described in the literature to produce some lignocellulolytic enzymes, such as β-galactosidases and endo-β-1,4-glucanases [15,16].

Due to the complex chemical structure of plant cell walls, the fractioning aiming generation of fibers, polysaccharides, polyphenols (lignin), and simple sugars from tamarind, jatoba, or other plants, is complex [17]. Microbial enzymes are widely valued for facilitating this process, as they allow the production of essential byproducts for biorefinery and bioenergy [18,19].

Among the microorganisms, filamentous fungi are fascinating mainly because they secrete enzymes and other primary and secondary metabolites to the culture medium during growth, in contrast to other organisms [6]. Therefore, the fungi prospection from the soil, humus, plant deterioration, etc., is an exciting approach to a systematic search involving novel microorganisms and enzymes with industrial applications [20,21]. Thus, it is hoped that the filamentous fungi are developed into great tools to find new solutions to society’s problems, especially those related to health, food, and bioenergy.

Alternatively, bioprospecting activities can motivate and help fund biodiversity conservation, especially in tropical regions, because of the rich biota they harbor [20,22]. This type of action can positively affect economic and social development, rewarding countries rich in genetic diversity but low in income, enabling the exploration and rational use of previously unknown resources [23].

This way, this study has the innovative nature of prospecting fungal lignocellulosic enzymes, never before explored through the microorganism species isolated for this study, which have industrial potential in the hydrolysis of biomass, as in the formulation of enzymatic cocktails used in the degradation of lignocellulosic residues. These enzymes are produced through a temporal analysis using seeds of jatoba and tamarind due to the abundance of xyloglucan in these carbon sources. The enzymatic production was staggered in a bioreactor, aiming at their fermentable sugar profiles. The enzymes were produced from different filamentous fungi collected and isolated on the campus of the University of São Paulo, Brazil, in spaces little explored by the community, but in the face of a forest, with rich soil for plants and decomposing materials. Therefore, the purpose of the critical methods of this study was the collection, isolation, and identification of microorganisms little explored in the literature, in addition to measuring the activity of enzymes used in the degradation of lignocellulosic biomass, such as sugarcane bagasse, where the saccharification profile was verified.

## 2. Material and Methods

### 2.1. Sampling and Isolation of Filamentous Fungi

The samples were collected from trunks of fifteen trees at the campus of the University of São Paulo, Ribeirão Preto, Brazil. At the collection, a fragment of approximately 2 cm² was taken from the tree trunk with the aid of a scalpel. The fragment was then were initially spread on a Petri dish containing the culture media oatmeal agar (4% oatmeal, 2% agar) [24] or wheat bran agar (2% wheat bran, 2% agar) [25]. Then, 1 mL of veterinary Pentabiotic (1 mg/mL) (Zoetis, Brazil) was added to both the culture media to prevent bacterial growth. The plates were incubated at 30 °C and 40 °C for seven days in a Biochemical Oxygen Demand (BOD) (Tecnal, Piracicaba, SP, Brazil) and monitored daily for fungal growth. For the next experiments, the fungi were grown only at the temperature which presented the best growth results. The different morphospecies grown on the plates were successively transferred to new plates until pure cultures were obtained. The fungal isolates were stored on agar slant tubes containing Potato Dextrose Agar (PDA) (Sigma-Aldrich, Saint Louis, MO, USA) and cryopreserved in glycerol 10% at −80 °C. All the isolated fungi were used in this study.

### 2.2. Plant Material

Sugarcane bagasse was provided by Pedra Agroindustrial S/A sugarcane mill (Serrana, SP, Brazil) and consists of a mixture of straw, leaves, and culm of several sugarcane kinds (CTC-4, CTC-7, CTC-20, IAC95500, RB867515, and RB966928). Energy cane culm was used from Centro de Cana—Instituto Agronômico (IAC), Ribeirão Preto, SP, Brazil, and mature sugarcane culms from kind SP80-3280 obtained by Centro de Ciências Agrárias—UFSCar, Araras, SP, Brazil.

These materials were sanitized by immersion in ethanol (92 °GL) for 1 h. The sugarcane was washed in distilled water afterward. The material was dried in the oven at 50 °C for 3 days and milled in a 25 mesh sieve knife mill (SL-32—SOLAB).

### 2.3. Fungal Identification 

#### 2.3.1. DNA Extraction

For molecular identification, the genomic DNA of the isolates was extracted through physical lysis of a mycelium disc. The mycelium was macerated in TES lysis buffer (100 mM Tris; 10 mM EDTA; 2% SDS) using a pestle and then incubated at 65 °C for 15 min. After this time, 140 µL of 5 M NaCl were added, and the samples were incubated on ice for 30 min. Then, 600 µL of chloroform:isoamyl alcohol (24:1) was added and centrifuged at 10,000× *g* for 10 min at 4 °C. The supernatant was collected, and 300 µL of isopropanol and 50 µL of 3 M sodium acetate buffer pH 5.2 were added. The material was centrifuged under the conditions above, and after discarding the supernatant, a new wash was performed with 600 µL of 70% ethanol, followed by a new centrifugation step. Finally, the supernatant was discarded and the precipitate diluted in 50 µL TE (10 mM Tris; 1 mM EDTA) with 5 µL RNAse (10 mg/mL) (ThermoFisher Scientific, Waltham, MA, USA) [22].

#### 2.3.2. Polymerase Chain Reaction Amplification 

After DNA extraction, the ITS region was amplified with the ITS4 and ITS5 [26]. Amplification reactions were performed using the PCR Master Mix kit (Promega, Madison, WI, USA) following the manufacturer’s instructions. The reaction product was later visualized in 1% agarose gel electrophoresis. The gel was stained with Nancy (Sigma-Aldrich, St. Louis, MO, USA) and detected on UV transilluminator. Purification of the amplification reaction was performed with the Wizard^®^ SV Gel and PCR Clean-up System Kit (Promega) and quantified in NanoDrop^®^ (Thermo Scientific, Waltham, MA, USA).

#### 2.3.3. DNA Sequencing 

The BigDye^®^ Terminator Cycle Sequencing Kit was used to sequence reactions (Life Technologies, Carlsbad, CA, USA) according to the manufacturer’s protocol and applied to ABI 3500XL sequencer (Life Technologies). The generated forward and reverse sequences were checked for quality and assembled into a consensus sequence using BioEdit v. 7.0.5.3 [27]. Contigs were compared with homologous sequences present in public databases such as NCBI-GenBank (www.ncbi.nlm.nih.gov) (accessed on 14 January 2021) through the BLASTn tool, and Trichokey database (http://isth.info/) (accessed on 14 January 2021). The sequences were subjected to second quality control and aligned with homologous sequences from culture collections using the ClustalW [28]. The sequences were subjected to phylogenetic analysis aiming to confirm taxonomic affiliation. The sequences were aligned with homologous sequences of phylogenetically related species using the ClustalW tool followed by manual refinement. The evolutionary history was inferred using the Neighbor-Joining method [29]. The optimal tree with a sum of branch length = 0.67745759 is shown. The percentage of replicated trees at which the associated taxa grouped in the bootstrap test (1000 replicates) is shown near branches [30]. The tree is drawn to scale, with branch lengths in the same units as the evolutionary distances used to infer the phylogenetic tree. The evolutionary distances were calculated using the 2-parameter Kimura method [31] and are in units of the number of base substitutions per location. Evolutionary analyses were conducted in MEGA 7.0 [32]. The nucleotide sequences were deposited at GenBank (Accession numbers MN249166-MN249172).

### 2.4. Selection of Microorganisms and Enzymatic Extract Preparations 

The screening of strains with higher enzymatic production levels was performed with the isolated filamentous fungi. For this objective, a solution with 10^6^–10^7^ spores/mL from the fungi was made. The fungi were grown in test tubes, suspended in sterile distilled water, and their spores were counted in a microscope through a Neubauer chamber. The suspension was inoculated into 125 mL Erlenmeyer flasks with 25 mL of Khanna medium (Khanna’s salt solution [20x]: NH_4_NO_3_ (2.0 g), KH_2_PO_4_ (1.3 g), MgSO_4_·7H_2_O (0.362 g), KCl (0.098 g), ZnSO_4_·H_2_O (0.007 g), MnSO_4_·H_2_O (0.0138 g), Fe_2_(SO_4_)_3_·6H_2_O (0.0066 g), CuSO_4_·5H_2_O (0.0062), distilled water q.s. (100mL) (5.0 mL); yeast extract (0.1 g); carbon source (1.0 g); distilled water q.s. (100 mL) [33]. The media were supplemented with 1% (*w/v*) of two different carbon sources, tamarind (*Tamarindus indica*, Fabaceae) or jatoba (*Hymenaea courbaril* L., Caesalpinioideae) seeds, which were previously pretreated (boiled in water, dried, and ground to 20 mesh) to secure the sanitary quality of the seeds and avoid the growth of other associated fungi. Erlenmeyer flasks were incubated at 30 °C under static or shaking (at 120 rpm) conditions, both up to 96 h, with sampling every 24 h. The samples were filtered with the aid of a vacuum pump, and the filtrates were used as enzymatic extracts to determine extracellular enzymatic activities, performed in triplicate.

### 2.5. Enzymatic Assays 

#### 2.5.1. Enzyme Determination Using Natural Substrates

The enzyme activities were measured with their respective substrates, which are cited between parentheses: endo-1,5-α-L-arabinanase (debranched arabinan) (Megazyme^®^), endoglucanase (carboxymethylcellulose—CMC) (Sigma-Aldrich^®^), β-glucanase (β-glucan) (Megazyme^®^), lichenase (lichenan) (Megazyme^®^), mannanase (locust bean) (Sigma-Aldrich^®^), polygalacturonase (polygalacturonic acid sodium salt) (Sigma-Aldrich^®^), endo-1,4-β-xylanase (xylan beechwood) (Sigma-Aldrich^®^), and xyloglucanase (xyloglucan) (Megazyme^®^). The activities were determined by the quantification of reducing sugars using the 3,5-dinitrosalicylic acid (DNS), according to the Miller method [34]. The assay mixture consisted of 25 µL of 1% (w/v) substrate solution in distilled water, 10 µL of 50 mM sodium acetate buffer, pH 5.0, and 15 µL of enzyme extract. A blank was carried out for each enzymatic assay by adding the enzyme extract after the assay time had elapsed with 50 µL of DNS. The absorbance was measured at 540 nm, and reducing sugars were quantified using standard curves of arabinose, cellobiose, glucose, mannose, galacturonic acid, and xylose (0–1 mg/mL), respectively. The detection limit was 3 µg of reducing sugar. The activity unit was defined as the amount of enzyme that releases one µmol of reducing sugar per minute per mL, and it was expressed as milliunity per mL (mU/mL).

#### 2.5.2. Enzyme Determination with Synthetic Substrates 

The enzymatic activities were measured with their respective substrates cited in parentheses: acetyl xylan esterase (*p*-nitrophenyl acetate) (Sigma-Aldrich^®^), α-L-arabinofuranosidase (*p*-nitrophenyl-α-L-arabinofuranoside) (Sigma-Aldrich^®^), β-D-galactosidase (*p*-nitrophenyl-β-D-galactopyranoside) (Sigma-Aldrich^®^), β-D-glucosidase (*p*-nitrophenyl-β-D-glycopyranoside) (Sigma-Aldrich^®^), β-D-xylosidase (*p*-nitrophenyl-β-D-xylanopyranoside) (Sigma-Aldrich^®^), and cellobiohydrolase (*p*-nitrophenyl-β-D-cellobioside) (Sigma-Aldrich^®^). The assay mixture consisted of 25 µL of 2 mM (w/v) substrate solution in distilled water, 10 µL of 50 mM sodium acetate buffer, pH 5.0, and 15 µL of the enzyme extract. The exception was acetyl xylan esterase, where the buffer used was 50 mM sodium citrate, pH 5.0; since the acetate buffer can be identified as a non-specific substrate for the enzyme [35]. After 20 min, 50 µL of Na_2_CO_3_ were added to interrupt the assay. A blank was performed for each enzymatic assay by adding the enzyme extract after the assay time had elapsed with 50 µL of Na_2_CO_3_ [36]. The absorbance was measured at 410 nm using a standard curve of *p*-nitrophenol (0–1 mg/mL). The detection limit was 3 µg. The activity unit was defined as the amount of enzyme that releases one µmol of *p*-nitrophenol per minute per mL, and it was expressed as milliunit per mL (mU/mL).

#### 2.5.3. Scaling for Bioreactor 

In order to obtain a more outstanding enzymatic production, cultivation was carried out in a 5 L BioFlo 310-New Brunswick^®^ bioreactor, containing 3.0 L of workload, under batch fermentation. The same culture medium previously used for cultivation in Erlenmeyer flasks was previously sterilized in an autoclave at 121 ° C for 30 min and aseptically placed in the reactor. The aeration of 1.0 vvm was performed by continuous injection of filtered compressed air of a sterile filter. The dissolved oxygen concentration (DO) was controlled, employing a DO probe ranging from 0 to 100%. A volume of 3 mL of antifoam 204 (Sigma^®^ A 6426) was added to the culture medium at the beginning of the process. The fermentation was carried out at 30 °C for *Trichoderma longibrachiatum* and 50 °C for *Thermothelomyces thermophiles*, for 72 h, using tamarind seeds were as carbon source. Protein concentration and DO were monitored every 24 h through an appropriate collector for the bioreactor, allowing the safe taking of samples.

The following formula was used to scale and determine that the agitation speed would be 280 rpm:

$$Ni\cdot tm=1.54\;V/Di^{3}$$

where,

*Ni* = stirring speed (1/s);
*tm* = mixing time constant;
*V* = volume of medium;
*Di* = impeller diameter.

#### 2.5.4. Protein Quantification

The proteins obtained in extracellular solutions were quantified by Bradford [37], where 40 µL of Bradford’s reagent were added to 160 µL of the enzymatic extracts and incubated for 5 min at room temperature. The absorbance was read on a spectrophotometer (Shimadzu, Kyoto, Japan) at 595 nm, using bovine albumin as standard. The results were expressed in μg of protein/mL.

#### 2.5.5. Pretreatment of Lignocellulosic Biomass

Sugarcane bagasse and culms from Energy cane (EC) and SP80-3280 sugarcane were pretreated by autohydrolysis and chemical treatments at 190 °C for 20 min. The pretreatment by autohydrolysis was carried out in a cylindrical stainless steel reactor (5.0 cm internal diameter and 12.8 cm internal height) with a working volume of 50 mL. Simultaneously, the solids loading rate was fixed at 10% (*w/v*), according to Michelin and Teixeira [38]. The reactor was immersed in an oil bath with an open heating circulator with temperature control. Afterward, the reactor was immediately cooled in an ice bath to quench the reaction. The resulting material was stored until use at 20 °C. In the chemical pretreatment, 1 g of dried and pulverized sugarcane bagasse or culm was incubated with 20 mL of 80% ethanol, at 80 °C, for 20 min to eliminate soluble sugars, and this procedure was repeated six times. The sample was centrifuged for 15 min (10,000× *g*), and the pellet was washed with 20 mL of distilled H_2_O and dried at 60 °C, overnight. After that, the material was incubated with 20 mL of 90% DMSO, for 24 h, at 90 °C for starch removal. The following pectin removal was carried out by setting the residual biomass with 20 mL of 0.5% ammonium oxalate, pH 7.0, at 80 °C, for 3 h. Finally, the delignification was performed by incubating the remaining bagasse with 20 mL of 0.5 M sodium chloride/acetic acid solution at 65 °C for 1 h, leaving only holocellulose, which consists of cellulose and hemicellulose [39].

#### 2.5.6. Enzymatic Hydrolysis and Determination of Sugars Content in Hydrolysates

The crude extracts, containing enzymes of the lignocellulolytic systems, were applied in assays with a final volume of 4 mL containing 3% (*w/v*) sugarcane bagasse (SCB), culm of energy cane (EC) or culm of sugarcane SP80-3280, 0.4 mL of 50 mM sodium acetate buffer, pH 5.0, and 3.6 mL of enzymatic extract. The experiments were conducted at 50 °C, under 200 rpm stirring, for 24 h. After this period, the materials were centrifuged (4800 rpm) at 4 °C. Sugar profiles of hydrolysates were determined using a High-Performance Liquid Chromatography (HPLC) YL9100 model (Young Lin Instruments) system equipped with the Rezex™ ROA-Organic Acid H^+^ (8%) 300 × 7.8 mm column and the YL9170 Refractive Index Detector. The analysis was performed at 80 °C, using 0.005 N H_2_SO_4_ as the mobile phase at a flow rate of 0.5 mL/min, with the detector cell temperature of 40 °C and a run time of 25 min. Xylose, cellobiose, arabinose, and glucose standards, at different concentrations, were dissolved in the mobile phase (0.005 N H_2_SO_4_) and analyzed at the same conditions of samples for the calibration.

### 2.6. Statistical Analysis

The experimental results were expressed by the mean ± standard deviation of three independent replicates using the program GraphPad Prism^®^ 5.0 (GraphPad Software, San Diego, CA, USA).

## 3. Results and Discussion

### 3.1. Identification of Microorganisms

**Figure 1:**
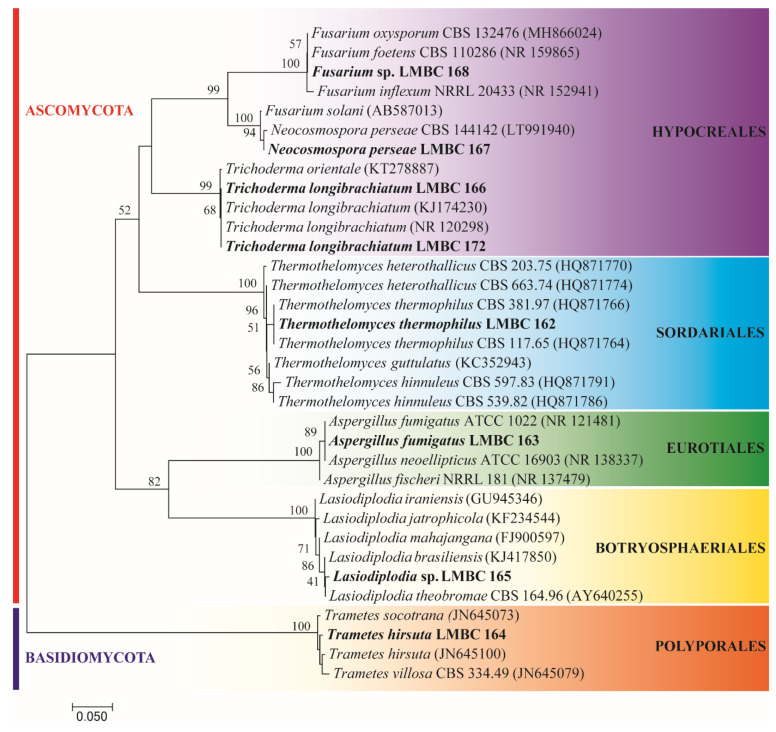
Phylogenetic relationship among the filamentous fungi isolated from tree trunks (bold) and the closest related species.

Fungi were isolated at the campus of the University of São Paulo, Ribeirão Preto, Brazil. The city has four main vegetation groups: mesophytic forests, deciduous forests, swampy forests, and cerrado [40]. All biomes have a significant number of jatoba and tamarind trees. Among the isolates, eight fungi were grown and maintained at laboratory conditions. The molecular identification showed high phylogenetic diversity (Figure 1), with representatives from the Phylum Ascomycota (*Fusarium* sp. LMBC 168; *Neocosmospora perseae* LMBC 167; *Trichoderma longibrachiatum* LMBC 166; LMBC 172; *Thermothelomyces thermophilus* LMBC 162; *Aspergillus fumigatus* LMBC 163, *Lasiodiplodia* sp. LMBC 165; and the Basidiomycota (*Trametes hirsuta* LMBC 164). LMBC stands for Laboratory of Microbiology and Cell Biology. This difference in phyla found represents the vast diversity of microorganisms found in biomes still little explored, wherein a small area.

### 3.2. Prospection of Microorganisms and Enzymatic Production 

**Figure 2:**
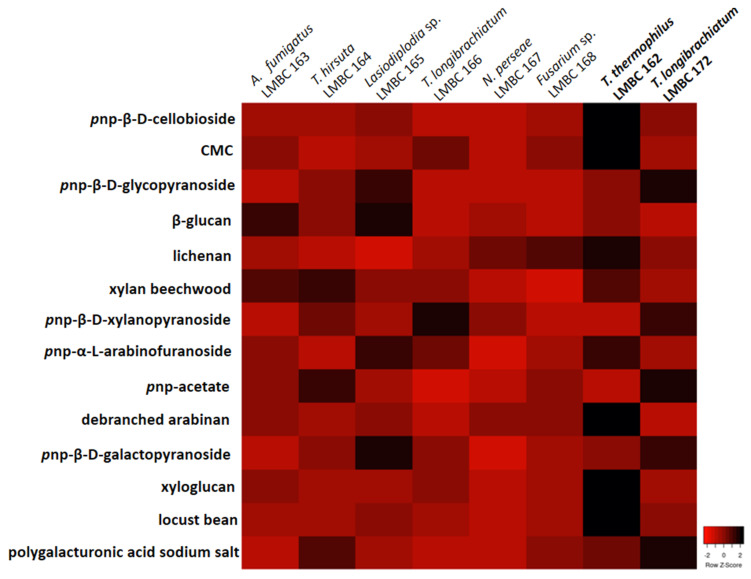
Heatmap regarding the eight isolates and the fourteen produced enzymes with their substrates.

This study’s next goal was to select among the eight identified fungi the best enzyme producers in a medium supplemented with tamarind seeds as a carbon source. A system of cultivation under agitation, for 72 h, was used. As observed in the heatmap (Figure 2), the principal enzyme producers were *T. thermophilus* LMBC 162 and *T. longibrachiatum* LMBC 172.

*T. thermophilus* efficiently hydrolyzed *p*np-cellobioside, CMC, lichenan, xylan, *p*np-arabinofuranoside, arabinan, xyloglucan, and locust bean. In contrast, *T. longibrachiatum* hydrolyzed *p*np-glycopyranoside, *p*np-xylanopyranoside, *p*np-acetate, *p*np-galactopyranoside, and polygalacturonic acid sodium salt, which means that it presented high enzymatic activity at the hydrolysis of the substrates above.

Interestingly, the other *T. longibrachiatum* isolated, referenced as LMBC 166, was one only good producer of *p*np-xylanopyranoside. A possible explanation for this phenomenon is attributed to the different collection sites. *T. longibrachiatum* LMBC 172 was isolated from decomposing plant material rich in lignocellulosic compounds. Instead, *T. longibrachiatum* LMBC 166 was collected from the trunks of living trees in good condition.

Time-course of the production of fourteen enzymes involved in the plant cell wall degradation from these two fungi, analyzing the influence of supplementary carbon sources (tamarind or jatoba) in the culture medium and the physical incubation conditions (cultures with and without agitation) was carried out as follows.

#### 3.2.1. Time-Course of Enzyme Production from *T. thermophilus* LMBC 162: Culture with Jatoba Seeds in Agitated and Static Conditions

Significant differences were noted comparing the two physical conditions of the culture of *T. thermophilus* LMBC 162 added with jatoba seeds. The great majority of the enzymes presented maximum activity at 48 h or 72 h under agitated conditions (Table 1). Exceptions were β-D-xylosidase, cellobiohydrolase, and mannanase secreted later, at 96 h.

Similarly, this fungus grown under static conditions showed maximum activity to the great majority of the enzymes studied in 48 and 72 h, but α-L-arabinofuranosidase, β-D-galactosidase, β-D-glucosidase, endoglucanase, and endo-1,4-β-xylanase were also secreted in maximal levels at 96 h.

The holoenzymes were produced with the most significant enzymatic activity in cultures under agitation compared with a static condition for most enzymes. The exceptions were arabinofuranosidase, endo-1,5-α-L-arabinanase, β-galactosidase, and xyloglucanase. The agitation processes showed advantages like the mixture of soluble components in the medium, dispersion of gases in the liquid medium, suspension of solid particles, and improved heat transfer [41].

#### 3.2.2. Time-Course of Enzyme Production from *T. thermophilus* LMBC 162: Culture with Tamarind Seeds in Agitated and Static Conditions

Mannanase and polygalacturonase were secreted, especially at 24 h when *T. thermophilus* was grown under agitated conditions. At the same time, cellobiohydrolase, endoglucanase, β-glucanase, lichenase, endoxylanase, β-D-xylosidase, α-L-arabinofuranosidase, and β-D-galactosidase were secreted, especially, between 48 h and 72 h. β-D-glucosidase, acetyl xylan esterase, and xyloglucanase had secreted at greater levels at 96 h (Table 2).

On the other hand, *T. thermophilus* grown in the culture medium added with tamarind seeds, under static conditions, showed that most of the enzymes were secreted to the culture media between 72 h and 96 h, with a different profile from the one verified under agitation. On the other hand, previous incubation periods (24 h) did not provide high extracellular levels.

Comparing the two physical conditions of development, when *T. thermophilus* was grown in the culture medium supplemented with tamarind seeds, similarly to what was verified for jatoba seeds, the fungus produced holoenzymes favorably in cultures under agitation with higher enzymatic levels (1.2–2.5-fold). On the other hand, endoglucanase, β-glucosidase, lichenase, β-xylosidase, and polygalacturonase had higher secretion levels in the cultures in static conditions (1.1–1.6-fold). Additionally, when cultivated under stationary conditions, it was observed that most enzymes are protagonists with more extended periods, showing a more significant delay in enzymatic production.

In conclusion, the best conditions for enzymatic secretion from *T. thermophilus* were in general cultures supplemented with jatoba seeds (Table 1), under agitation, during 48–72 h. Tamarind seeds were potential inducers for acetyl xylan esterase (Table 2) compared with jatoba seeds. According to Pereira [42], the tamarind composition is 6.26% ± 0.08 lignin, 5.79 ± 0.60 cellulose, and 17.32 ± 0.26 hemicellulose, while the composition of jatoba is 59.78% holocellulose, 28.7% lignin, 13.32% extractives, and 0.48% ashes [43].

#### 3.2.3. Time-Course of Enzyme Production from *T. longibrachiatum* LMBC 172: Culture with Jatoba Seeds in Agitated and Static Conditions

Regarding the isolated *T. longibrachiatum* LMBC 172, when it was cultivated with jatoba seeds, under agitation, the time of 24 h was not significant for any enzyme. Still, cellobiohydrolase, endoglucanase, and mannanase were secreted at 48 h, while α-L-arabinofuranosidase and polygalacturonase were secreted later, at 96 h (Table 3). The secretion of the other nine enzymes studied was more significant at 72 h.

Under static conditions, *T. longibrachiatum* showed different behavior. β-glucanase was the first to be secreted at 24 h of cultivation, in contrast to mannanase and β-glucosidase, which were secreted later, at 96 h. Three enzymes were secreted with maximum activity at 48 h: endoglucanase, acetyl xylan esterase, and endo-1,5-α-arabinanase. The other reached their maximum secretion at 72 h. These results demonstrate that the cultivation conditions positively influence the enzymatic secretion in addition to the carbon source.

Regarding the results, it is possible to conclude that when the two physical conditions of development for *T. longibrachiatum* growth in jatoba seed-culture medium were compared, the static cultures permitted maximum levels to the majority enzymes (1.13–3.12-fold), i.e., endoglucanase, lichenase, endoxylanase, β-xylosidase, α-arabinofuranosidase, xyloglucanase, mannanase, and polygalacturonase. The secretion of cellobiohydrolase, β-glucosidase, and acetyl xylan esterase was satisfactorily verified under agitation (1.65–8.8-fold), probably due to the better aeration.

#### 3.2.4. Time-Course of Enzyme Production from *T. longibrachiatum* LMBC 172: Culture with Tamarind Seeds in Agitated and Static Conditions

It was shown that the great majority of enzymes when cultivated with tamarind seeds, under agitated conditions, were more effectively produced with 72 h (Table 4). β-D- glucosidase and cellobiohydrolase were exceptions, with the best time-course being 48 h. The progress of endoglucanase and xyloglucanase was at 96 h. However, in static cultivation, a different behavior was observed, where endo-1,5-α-arabinanase was the first enzyme secreted at a maximal level (24 h). Acetyl xylan esterase, lichenase, and polygalacturonase presented the best time to secretion at 48 h. The other enzymes were secreted at high levels, mainly at 72 h–96 h.

Comparing the two physical conditions of development (Table 3 and Table 4), when *T. longibrachiatum* was grown in a tamarind seed-culture medium (Table 4), the fungus favorably produced holoenzymes in cultures under agitation. Higher enzymatic levels (1.1–6.7) of the cellulolytic enzymes (cellobiohydrolase, β-glucosidase, β-glucanase, and lichenase, except endoglucanase) besides endo-1,5-α-arabinanase, mannanase, and pectinase activities were observed. Secretion of xylanolytic enzymes, as endoxylanase, β-xylosidase, α-arabinofuranosidase, xyloglucanase, and β-galactosidase was favored to cultivation without agitation. Acetyl xylan esterase was secreted at equal levels in both conditions.

In general, the best conditions for enzyme secretion from *T. longibrachiatum* were tamarind seed-cultures, under agitation, for 72 h (Table 4). Exceptions were observed for lichenase, acetyl xylan esterase, and β-galactosidase because these enzymes were preferentially secreted when jatoba seeds had supplemented the culture medium (Table 3).

#### 3.2.5. Scaling for the Bioreactor to Increase Enzyme Production

The bioreactor scaling allowed the comparison of total protein production in Erlenmeyer flasks and the 5 L BioFlo 310-New Brunswick^®^ bioreactor for 72 h using tamarind seeds as a carbon source. A verified increase of 70.5% was seen for *T. longibrachiatum* in the secretion of proteins. In comparison, an increase of 74.5% was verified for *T. thermophilus*, as seen in Table 5. This result demonstrated the advantages of scaling up to improve the protein concentration. Consequently, this fact may have generated a more significant amount of enzymes, favoring enzymatic hydrolysis in lignocellulosic residues in the subsequent phase.

#### 3.2.6. Sugar Profile

**Figure 3:**
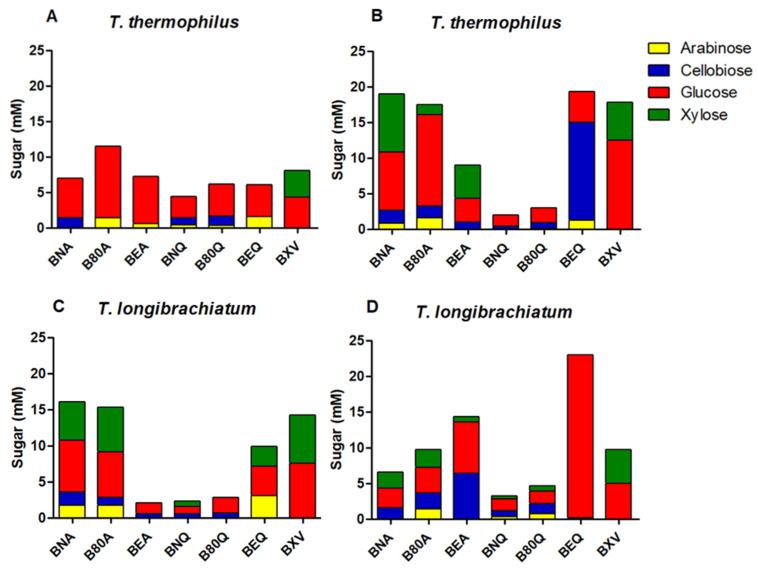
Sugars content in hydrolysates. **BNA:** sugarcane bagasse pretreated by autohydrolysis; **B80A:** SP80-3280 sugarcane culm pretreated by autohydrolysis; **BEA:** culms of energy cane pretreated by autohydrolysis; **BNQ:** sugarcane bagasse pretreated chemically; **B80Q:** SP80-3280 sugarcane culm pretreated chemically; **BEQ:** culms of energy cane pretreated chemically; **BXV:** steam explosion sugarcane bagasse. (**A**,**B**) values of *T. thermophiles*; (**C**,**D**) values of *T. longibrachiatum*.

The hydrolysis products of *T. thermophilus* crude enzyme extracts cultivated in jatoba seeds on pretreated sugarcane bagasse are represented in Figure 3A. The sugarcane and pretreatments used were: sugarcane bagasse pretreated by autohydrolysis (BNA), SP80-3280 culm sugarcane pretreated by autohydrolysis (B80A), culms of energy cane pretreated by autohydrolysis (BEA), sugarcane bagasse pretreated chemically (BNQ), SP80-3280 culm sugarcane pretreated chemically (B80Q), culms of energy cane pretreated chemically (BEQ), and steam explosion sugarcane bagasse (BXV). A predominance of glucose was found in all samples, with xylose being detected only in BXV. A hypothesis for the non-identification of xylose can be justified by the enzymatic extract being rich in hemicellulases that degrade xyloglucan, which proves the high amount of glucose (product of hydrolysis of xyloglucan) when compared with xylose [44].

In Figure 3B, the sugar profile obtained from hydrolysis of crude extracts of *T. thermophilus* cultivated in tamarind seeds on various sugar cane samples can be seen. Similarly, glucose was predominant in all pretreatments, except in BEQ, in which cellobiose was the sugar found in higher quantity. This fact may be due to the lower β-glucosidase levels obtained in this condition.

Figure 3C shows the cultivation of *T. longibrachiatum* in jatoba seeds. Glucose was also the sugar found in greater quantity in this case. Still, arabinose, especially on BNA, B80A, and BEQ, was also a meaningful result. However, the highest sugar content is seen in Figure 3D, when crude extracts of *T. longibrachiatum* grown in tamarind seeds were used for BEQ hydrolysis, reaching a quantity of almost 25 mM of glucose. The hydrolysis of tamarind seed-crude extract on all sugar cane residues resulted in addition to glucose, large amounts of arabinose, cellobiose, and xylose.

## 4. Conclusions

This study permits us to conclude that microorganisms and enzymes prospected are essential to finding a good producer of lignocellulosic enzymes with exciting industrial applications. Eight fungi were isolated, belonging to the phylum Ascomycota and Basidiomycota. Among these, *T. thermophilus* and *T. longibrachiatum* have shown effectiveness in the secretion of fourteen enzymes, in special β-glucanase, endo-1,4-β-xylanase, and xyloglucanase. These enzymes are of economic interest in the degradation of biomass. They were produced using a temporal analysis (24 to 96 h) with residues of plants rich in xyloglucans, like tamarind and jatoba seeds, but 72 h was considered an optimal time for enzyme secretion. From a practical point of view, the high levels of enzymatic activity produced by the microorganisms isolated in this study demonstrate its applicability compared to other reports in the literature. Regarding the formation of reducing sugar, tamarind seeds were the best option for both *T. thermophilus* and *T. longibrachiatum,* but not for *T. thermophilus* enzyme production. The kind of sugarcane, the pretreatment used, the choice of microorganisms, physical conditions of cultivation, and the carbon sources proved to be limiting factors in the sugar profile resulting from biomass hydrolysis.

## Figures and Tables

**Table 1:** Enzymatic activities of *Thermothelomyces thermophilus* LMBC 162 cultivated with jatoba seeds during different times.

Enzyme	Under Agitation				Static			
	24 h	48 h	72 h	96 h	24 h	48 h	72 h	96 h
***cellobiohydrolase***	4.6 ± 0	19.7 ± 1.2	25.0 ± 1.0	**33.0 ± 2.0**	2.1 ± 0	10.7 ± 0.6	**17.3 ± 2.5**	7.7 ± 0.9
***endoglucanase***	8.0 ± 1.7	**107.3 ± 4.6**	65.0 ± 9.5	53.0 ± 1.7	17.0 ± 2.0	77.3 ± 3.8	78.7 ± 0.6	**84.3 ± 3.5**
***β-D-glucosidase***	6.0 ± 0.3	51.0 ± 0	**73.0 ± 0.6**	69.0 ± 0.9	5.7 ± 0.3	11.0 ± 1.7	24.3 ± 2.9	**25.0 ± 1.0**
***β-glucanase***	32.0 ± 4.6	**159.3 ± 6.7**	93.6 ± 3.1	75.3 ± 11.9	5.7 ± 0.9	57.0 ± 9.5	**109.0 ± 7.9**	71.0 ± 11.1
***lichenase***	70.3 ± 9.5	**299.0 ± 8.2**	192.7 ± 9.5	90.3 ± 11.0	157.0 ± 0.9	**245.0 ± 24.6**	159.7 ± 3.5	145.3 ± 10.1
***endo-1,4-β-xylanase***	218.7 ± 44.5	893.7 ± 69.4	**1043.3 ± 28.0**	886.3 ± 20.6	31.0 ± 1.0	722.0 ± 12.5	845.3 ± 3.7	**936.7 ± 1.2**
***β-D-xylosidase***	3.0 ± 0.3	3.7 ± 0.4	4.8 ± 0	**4.9 ± 0.5**	**3.1 ± 0.4**	2.7 ± 0	2.0 ± 0.4	1.9 ± 0.4
***α-L-arabinofuranosidase***	3.5 ± 0.2	4.9 ± 1.1	**7.0 ± 0.8**	nd	1.5 ± 0.5	2.9 ± 0.2	5.9 ± 1.1	**9.0 ± 1.2**
***acetyl xylan esterase***	nd	**19.0 ± 2.0**	15.0 ± 1.0	5.3 ± 1.9	nd	1.6 ± 0.9	**15.7 ± 1.2**	4.2 ± 1.6
***endo-1,5-α-L-arabinanase***	81.0 ± 6.2	**130.3 ± 16.1**	105.0 ± 1.0	95.3 ± 2.5	55.5 ± 3.5	**180.0 ± 6.2**	158.0 ± 4.6	71.3 ± 4.0
***β-D-galactosidase***	nd	6.1 ± 0	**11.3 ± 0.6**	9.6 ± 1.2	2.7 ± 0.3	10.3 ± 0.6	15.0 ± 0	**24.0 ± 1.0**
***xyloglucanase***	22.3 ± 2.6	180.7 ± 18.2	**210.0 ± 13.1**	138.0 ± 18.0	nd	177.7 ± 3.5	**311.0 ± 9.5**	274.3 ± 5.1
***mannanase***	179.7 ± 30.0	**265.0 ± 25.9**	163.3 ± 7.2	**112.7 ± 4.0**	184.3 ± 1.2	**247.3 ± 6.4**	236.0 ± 2.6	226.7 ± 8.7
***polygalacturonase***	117.7 ± 0.6	133.3 ± 7.2	**213.7 ± 4.0**	56.7 ± 7.8	24.3 ± 4.2	**138.7 ± 14.3**	100.0 ± 6.0	88.7 ± 9.7

Values expressed in mU/mL; nd = not detected. In bold, the maximum value was found for each enzyme.

**Table 2:** Enzymatic activities of *Thermothelomyces thermophilus* LMBC 162 cultivated with tamarind seeds during different times.

Enzyme	Under Agitation				Static			
	24 h	48 h	72 h	96 h	24 h	48 h	72 h	96 h
***cellobiohydrolase***	2.3 ± 0.5	4.0 ± 0.6	**5.0 ± 0.3**	3.8 ± 0.7	1.4 ± 0	3.1 ± 0.3	**3.4 ± 0.2**	nd
***endoglucanase***	23.0 ± 10.6	**39.3 ± 6.7**	36.7 ± 5.0	23.0 ± 10.1	27.0 ± 4.4	**43.7 ± 8.7**	38.3 ± 1.2	27.7 ± 4.2
***β-D-glucosidase***	0.9 ± 0.5	11.7 ± 0.6	13.3 ± 1.5	**16.7 ± 1.2**	7.3 ± 0.7	19.0 ± 1.0	**26.7 ± 0.6**	19.3 ± 1.2
***β-glucanase***	21.0 ± 1.5	103.0 ± 8.2	**136.7 ± 6.1**	118.3 ± 6.1	25.7 ± 3.2	41.3 ± 5.7	49.3 ± 1.5	**66.3 ± 7.6**
***lichenase***	nd	7.0 ± 3.1	**86.3 ± 12.0**	nd	73.7 ± 19.3	**113.0 ± 6.9**	101.7 ± 21.7	58.7 ± 2.5
***endo-1,4-β-xylanase***	372.0 ± 12.1	649.3 ± 9.7	**862.0 ± 10.8**	660.0 ± 33.4	127.3 ± 3.9	363.7 ± 5.9	550.0 ± 8.9	**701.7 ± 62.9**
***β-D-xylosidase***	1.6 ± 0.5	**3.1 ± 0.5**	**3.1 ± 1.0**	1.4 ± 0.2	1.2 ± 0.4	2.1 ± 0.5	3.9 ± 1.3	**4.3 ± 0.7**
***α-L-arabinofuranosidase***	0.9 ± 0.1	**5.3 ± 0.3**	4.4 ± 0.2	3.6 ± 0.3	1.7 ± 0.8	2.3 ± 0.6	1.5 ± 0.9	**4.4 ± 0.7**
***acetyl xylan esterase***	nd	13.3 ± 2.3	19.0 ± 1.0	**47.0 ± 0.7**	nd	2.6 ± 0.9	39.0 ± 3.0	**43.3 ± 0.6**
***endo-1,5-α-L-arabinanase***	27.3 ± 10.0	51.7 ± 13.4	**86.0 ± 13.2**	23.3 ± 9.6	30.3 ± 7.5	34.3 ± 7.6	**34.7 ± 9.9**	9.3 ± 0.7
***β-D-galactosidase***	2.5 ± 0.4	**3.0 ± 0.1**	2.6 ± 0.4	1.8 ± 0.4	0.5 ± 0.2	**1.0 ± 0.2**	**1.0 ± 0.2**	0.9 ± 0.5
***xyloglucanase***	11.7 ± 7.6	46.3 ± 5.5	74.0 ± 4.6	**84.0 ± 9.5**	18.7 ± 2.1	39.3 ± 6.0	**51.3 ± 0.6**	27.3 ± 6.5
***mannanase***	**77.0 ± 5.3**	54.3 ± 4.7	52.3 ± 2.6	1.3 ± 0.3	28.7 ± 4.9	**56.0 ± 7.1**	29.7 ± 6.1	35.7 ± 4.0
***polygalacturonase***	**81.7 ± 6.4**	54.0 ± 5.0	22.0 ± 2.4	23.0 ± 4.2	26.7 ± 4.0	68.5 ± 6.4	**85.7 ± 11.0**	58.3 ± 1.5

Values expressed in mU/mL; nd = not detected. In bold, the maximum value was found for each enzyme.

**Table 3:** Enzymatic activities of *Trichoderma longibrachiatum* LMBC 172 cultivated with jatoba seeds during different times.

Enzyme	Under Agitation				Static			
	24 h	48 h	72 h	96 h	24 h	48 h	72 h	96 h
***cellobiohydrolase***	2.3 ± 0.2	**3.8 ± 1.0**	1.4 ± 0.7	0.5 ± 0.2	0.6 ± 0.3	1.4 ± 0.4	**2.3 ± 0.5**	1.1 ± 0.1
***endoglucanase***	11.0 ± 4.4	**54.7 ± 4.7**	45.7 ± 7.5	18.7 ± 6.5	24.7 ± 1.5	**67.3 ± 0.6**	50.7 ± 11.1	50.3 ± 9.8
***β-D-glucosidase***	9.3 ± 2.3	10.0 ± 0	**11.0 ± 1.0**	8.7 ± 1.2	3.4 ± 0.2	5.1 ± 0.2	6.3 ± 0.3	**7.0 ± 0.6**
***β-glucanase***	103.0 ± 1.5	177.0 ± 14.1	**223.7 ± 19.1**	59.3 ± 6.7	**231.3 ± 20.5**	161.7 ± 5.8	129.7 ± 10.5	41.3 ± 9.0
***lichenase***	70.3 ± 5.5	75.5 ± 10.5	**97.3 ± 4.0**	63.3 ± 17.0	131.3 ± 30.9	227.0 ± 5.8	**244.0 ± 42.0**	78.7 ± 17.0
***endo-1,4-β-xylanase***	30.0 ± 6.4	429.3 ± 9.5	**492.3 ± 6.5**	462.0 ± 4.4	31.0 ± 1.8	385.3 ± 33.0	**772.3 ± 35.1**	705.0 ± 23.8
***β-D-xylosidase***	1.2 ± 0	8.1 ± 0.9	**10.0 ± 1.0**	9.7 ± 1.2	nd	5.7 ± 0.2	**19.0 ± 1.0**	18.3 ± 2.1
***α-L-arabinofuranosidase***	1.2 ± 0.5	3.7 ± 0.3	4.9 ± 0.3	**6.2 ± 0.5**	1.6 ± 0.5	2.9 ± 0.2	**8.7 ± 0.6**	7.7 ± 0.6
***acetyl xylan esterase***	1.1 ± 0.1	7.1 ± 0.5	**53.7 ± 2.9**	8.6 ± 1.6	nd	**6.1 ± 0.6**	nd	nd
***endo-1,5-α-L-arabinanase***	nd	17.7 ± 5.1	**32.7 ± 8.1**	nd	3.3 ± 0.8	**18.0 ± 7.9**	1.8 ± 0.9	nd
***β-D-galactosidase***	2.3 ± 0.3	7.0 ± 0.7	**9.3 ± 0.6**	7.4 ± 0.7	2.8 ± 0.2	3.8 ± 0.4	**9.0 ± 0**	7.2 ± 1.8
***xyloglucanase***	11.7 ± 3.5	45.0 ± 7.2	**61.7 ± 4.0**	40.0 ± 5.3	14.1 ± 8.2	14.0 ± 7.2	**69.7 ± 9.6**	67.7 ± 12.3
***mannanase***	69.7 ± 3.1	**99.7 ± 3.9**	64.7 ± 10.5	2.7 ± 0.6	10.0 ± 3.6	71.0 ± 3.6	79.3 ± 5.9	**102.7 ± 9.5**
***polygalacturonase***	3.0 ± 0.8	4.0 ± 0.4	7.0 ± 1.2	**8.1 ± 0.6**	4.0 ± 0.9	7.3 ± 0.7	**25.3 ± 18.5**	10.5 ± 4.9

Values expressed in mU/mL; nd = not detected. In bold, the maximum value was found for each enzyme.

**Table 4:** Enzymatic activities of *Trichoderma longibrachiatum* LMBC 172 cultivated with tamarind seeds during different times.

Enzyme	Under Agitation				Static			
	24 h	48 h	72 h	96 h	24 h	48 h	72 h	96 h
***cellobiohydrolase***	3.5 ± 0.9	**16.7 ± 1.2**	1.8 ± 0.6	2.1 ± 0.7	0.3 ± 0.1	1.4 ± 0.4	**2.5 ± 0.3**	1.4 ± 1.1
***endoglucanase***	20.7 ± 3.2	33.0 ± 5.6	43.0 ± 5.1	**50.7 ± 1.5**	nd	44.0 ± 4.6	**84.3 ± 10.7**	61.7 ± 14.7
***β-D-glucosidase***	16.0 ± 0	**42.7 ± 2.1**	38.3 ± 1.5	23.7 ± 2.3	1.9 ± 0.3	8.2 ± 1.3	**24.0 ± 2.6**	16.3 ± 2.1
***β-glucanase***	181.7 ± 40.2	236.3 ± 19.4	**389.0 ± 2.0**	244.0 ± 6.0	22.7 ± 8.3	125.0 ± 9.5	185.7 ± 6.4	**203.0 ± 11.1**
***lichenase***	30.0 ± 10.4	60.3 ± 6.1	**94.7 ± 32.9**	84.0 ± 10.4	3.0 ± 0.9	**36.0 ± 3.6**	1.3 ± 0.3	nd
***endo-1,4-β-xylanase***	64.0 ± 9.8	225.3 ± 28.5	**805.0 ± 32.7**	725.0 ± 49.0	74.7 ± 2.5	776.0 ± 6.8	**1011.0 ± 70.4**	933.7 ± 26.7
***β-D-xylosidase***	2.4 ± 0.6	3.9 ± 0.1	**12.3 ± 1.2**	7.4 ± 0.7	0.9 ± 0.2	4.6 ± 0.2	5.0 ± 0.4	**21.7 ± 3.8**
***α-L-arabinofuranosidase***	1.5 ± 0.8	2.5 ± 0.1	**4.1 ± 0.7**	nd	0.3 ± 0.1	1.4 ± 0.8	2.6 ± 0.3	**10.7 ± 1.2**
***acetyl xylan esterase***	0.02 ± 0	nd	**10.3 ± 1.5**	7.2 ± 0.8	0.3 ± 0.1	**10.0 ± 1.0**	0.6 ± 0.2	nd
***endo-1,5-α-L-arabinanase***	27.3 ± 10.0	51.7 ± 13.4	**86.0 ± 13.2**	23.3 ± 9.6	**27.0 ± 5.3**	21.0 ± 83	6.0 ± 0.6	nd
***β-D-galactosidase***	0.8 ± 0.4	5.6 ± 0.4	**6.7 ± 0.5**	15.0 ± 0	0.8 ± 0	3.0 ± 0.3	5.9 ± 1.3	**7.3 ± 0.4**
***xyloglucanase***	13.0 ± 2.8	20.0 ± 5.7	40.0 ± 6.8	**57.7 ± 4.3**	12.3 ± 3.2	188.7 ± 9.0	**325.7 ± 27.3**	194.3 ± 9.0
***mannanase***	63.7 ± 6.1	100.0 ± 5.6	**158.3 ± 18.9**	35.0 ± 6.6	74.7 ± 4.0	98.0 ± 18.2	**141.3 ± 18.9**	39.3 ± 10.0
***polygalacturonase***	71.0 ± 6.0	246.0 ± 5.6	**409.0 ± 18.0**	60.0 ± 16.0	27.3 ± 2.8	**352.0 ± 38.0**	218.0 ± 9.2	190.3 ± 14.0

Values expressed in mU/mL; nd = not detected. In bold, the found maximum value for each enzyme.

**Table 5:** Comparison between production in Erlenmeyer flasks and bioreactor.

	Temperature Growth (°C)	Total Proteins Erlenmeyer Flasks *	Total Proteins Bioreactor *
*T. longibrachiatum*	30	18.2 ± 1.57	25.8 ± 1.91
*T. thermophilus*	50	21.3 ± 1.64	28.6 ± 2.03

* Total proteins expressed in μg/mL.

## Data Availability

Not applicable.
